# High-load terephthalic acid degradation and diverse bioproduct formation by novel *Rhodococcus* strains

**DOI:** 10.1007/s10529-026-03739-z

**Published:** 2026-05-13

**Authors:** Caio Issamu Somiza, Nívea Moreira Vieira, Alex Gazolla de Castro, Isabela Pereira da Silva Bento, Kleryton Luiz Alves de Oliveira, Lívia Moreira Couto, Jomar de Lima Barros, Camila de Souza Vieira, Wendel Batista da Silveira, Renata Pereira Lopes Moreira, Marcos Rogério Tótola

**Affiliations:** 1 Department of Microbiology, Federal University of Viçosa, Viçosa, Brazil; 2 Department of General Biology, Federal University of Viçosa, Viçosa, Brazil; 3 Department of Chemistry, Federal University of Viçosa, Viçosa, Brazil

**Keywords:** Terephthalic acid, PET upcycling, Bioconversion, *Rhodococcus*

## Abstract

Terephthalic acid (TPA) from poly(ethylene terephthalate) (PET) depolymerization is a promising substrate for microbial upcycling, yet complete degradation at high concentrations has not been reported above 120 mM. Here, we report seven *Rhodococcus* strains isolated from compost enrichment cultures using TPA as the sole carbon source. Three isolates completely consumed 240 mM TPA in minimal medium under high osmolarity conditions, surpassing the previously reported threshold. Strain TA18 produced β-linked exopolysaccharides and a polyhydroxyalkanoate composed mainly of 3-hydroxyvalerate from TPA as the sole carbon source, neither of which has been previously described from this substrate in *Rhodococcus*, alongside C16 and C18 fatty acids. These strains expand the set of robust TPA-assimilating bacteria available for PET upcycling and provide a foundation for developing routes that convert plastic waste streams into multiple microbial products.

**Supplementary Information:**

The online version contains supplementary material available at 10.1007/s10529-026-03739-z.

## Introduction

Plastics are extensively used across modern society, but inadequate disposal and leakage from production cycles have led to widespread environmental contamination (Geyer et al. 2017). Recycling remains the main strategy for reducing plastic accumulation and demand for virgin fossil-based materials. Among plastics, poly (ethylene terephthalate) (PET), widely used in packaging and textiles, achieves the highest recycling rate, reaching 23% in 2020 compared to 9% for plastics overall (Duan et al. 2024). Yet recycling neither addresses the current reliance on persistent synthetic polymers nor provides a sustainable end-of-life solution for plastics already in circulation, which remain susceptible to environmental leakage. Chemical recycling offers a complementary path, as PET monomer recovery yielding terephthalic acid (TPA) and ethylene glycol (EG) can be economically viable, with TPA produced via mechanochemical depolymerization estimated at ~ $0.99/kg versus ~ $1.14/kg for TPA derived from petroleum. The economic margin, however, is modest and highly sensitive to feedstock costs and downstream purification steps (Anglou et al. 2024). An alternative strategy is to redirect PET waste into other production chains. Monomers released by chemical or enzymatic depolymerization can serve as microbial feedstocks, integrating waste treatment with sustainable bioproduction, potentially improving the economic incentives for plastic waste management (Qi et al. 2022).

Of the two monomers, TPA accounts for ~ 83% of PET mass and, unlike EG, which is readily converted into glycolic acid by a wide range of microorganisms (Carniel et al. 2024), its catabolism is restricted to bacteria harboring specialized aromatic degradation pathways. Microbial TPA assimilation is initiated by conversion to protocatechuate, which is then channeled through one of three ring-cleavage pathways generating central metabolic intermediates. As a result, TPA catabolism is restricted to a narrower set of bacteria, with *Comamonas*, *Pseudomonas*, and *Rhodococcus* being the most frequently employed genera in bioupcycling studies (Somiza et al. 2026). Genetic engineering has also been applied to expand the range of products obtained from TPA. For instance, an engineered *Pseudomonas putida* KT2440 converted BHET-derived TPA into β-ketoadipate, though complete consumption of the available 120 mM TPA was not achieved (Werner et al. 2021). Among wild-type strains, *Rhodococcus erythropolis* has been reported to completely consume 120 mM TPA within 84 h in optimized conditions, with PHA accumulation confirmed by FTIR (Maurya et al. 2024), representing the highest concentration of complete TPA degradation reported to date.

Here, we report seven *Rhodococcus* strains isolated from compost enrichment cultures using TPA as the sole carbon source. Three of these isolates completely consumed 240 mM TPA in minimal medium. Furthermore, strain TA18 produced exopolysaccharides, lipids, and polyhydroxyalkanoates (PHAs) mainly composed of 3-hydroxyvalerate from TPA as the sole carbon source. These findings expand the set of wild-type *Rhodococcus* strains available for PET upcycling, demonstrating both high-load TPA catabolism and the ability to channel TPA into biotechnologically relevant metabolites, supporting their potential as platforms for biological valorization of PET waste streams.

## Material and methods

### Screening of terephthalic acid–consuming isolates

Isolates derived from TPA-supplemented enrichment cultures inoculated with eucalyptus leaf and manure compost were cultured in MSM5 at 30 and 50 ºC. See Table S1 for media composition. Microbial growth was monitored by measuring OD_600_. After 72 h of incubation, residual TPA concentration in culture medium was quantified by high-performance liquid chromatography (HPLC). Isolates were classified according to their relative TPA consumption after 72 h of cultivation (Table S2). Strains exhibiting TPA consumption above 90% were selected for taxonomic identification and subsequent cultivation at a higher TPA concentration. Enrichment cultures preparation and HPLC method are fully described in Supplementary Methods (SM) 1 and 2, respectively.

Selected isolates were further cultivated in mineral salts medium supplemented with 240 mM TPA (MSM40). To fully solubilize 240 mM TPA, 450 mM NaOH was added to MSM40 prior to inoculation, generating disodium terephthalate (Na_2_TPA), which exhibits higher solubility (~ 130 g L^−1^). HEPES buffer (110 g L⁻^1^) was supplemented to maintain pH stability throughout the incubation period. Growth was monitored in triplicate by measuring OD_600_ at regular intervals until cessation, and residual TPA concentration in the culture medium was quantified by HPLC.

### Bacterial identification and strain separation

Sherlock Microbial Identification System (MIDI, Inc., USA) (MIDI) was used to determine whether the isolates corresponded to distinct strains. Fatty acid methyl ester (FAME) profiles were compared, and a dendrogram based on Euclidean distance was generated using the system software. A cutoff was applied at 2.5 to classify samples as belonging to the same isolate (ED < 2.5) or to different isolates (ED > 2.5). Fatty acid methyl esters were obtained and analyzed according to the suggested protocol of MIDI using a 7890 A gas chromatograph (Agilent Technologies), as described by Schutter and Dick (2000). Briefly, approximately 5 mg of wet biomass were subjected to saponification using a solution of NaOH with methanol to convert cell lipids into sodium salts in a water bath at 100 °C. To obtain FAMEs, fatty acids were derivatized using acidified methanol (HCl:MeOH). After the second heating, a mixture of hexadecane and methyl-*tert* butyl ether was added for organic and aqueous phase separation, and the organic phase containing FAMEs was collected for analysis.

For identification, partial 16S rRNA gene sequences were obtained by Sanger sequencing, deposited in the GenBank database (Table S3) and compared by BLASTn. A total of 14 sequences were included in the phylogenetic analysis between the isolated strains and reference sequences. The latter were selected based on BLASTn results, including type strains of the closest-matching group and species within that group previously reported to degrade TPA. Full molecular methods and phylogenetic parameters are available in SM3.

### Biosurfactant production assay

Isolates were cultivated in TSB and in mineral salts medium supplemented separately with 60 mM TPA (MSM10), soybean oil (20 g L⁻^1^), or n-hexadecane (20 g L⁻^1^). Cultures were incubated at 30 °C for 168 h under orbital shaking at 200 rpm. After incubation, culture samples were centrifuged, and the resulting cell-free supernatants were evaluated for biosurfactant production using the oil-spreading assay. Briefly, 15 µL of crude oil were layered onto 40 mL of distilled water in a Petri dish. After formation of a uniform oil film, 10 µL of culture supernatant were added to the oil surface. Biosurfactant production was indicated by the formation of a clear oil-spreading halo, as described by Morikawa et al. (1993).

### Exopolysaccharide production assay

To evaluate exopolysaccharide (EPS) production, isolates were cultivated on MSM10 agar supplemented with calcofluor white (0.2 g L⁻^1^) and incubated at 30 °C for 72 h. Exopolysaccharide production was indicated by colony fluorescence under ultraviolet light (365 nm) (Leigh et al. 1985).

### Polyhydroxyalkanoate production assay

Polyhydroxyalkanoate (PHA) production was initially screened by cultivating isolates on MSM10 agar for 72 h at 30 °C. For all media used for PHA production, the nitrogen source concentration was reduced by half (0.25 g L⁻^1^ NH_4_Cl) to promote carbon flux toward storage compound accumulation. Colonies were stained with a Nile blue sulfate solution in ethanol (0.5 g L⁻^1^), and PHA accumulation was indicated by fluorescence under ultraviolet light (365 nm) (Kitamura and Doi 1994).

Comparison of PHA production was performed using a microplate assay adapted from Oshiki et al. (2011). Selected isolates were cultivated in 200 µL of MSM10 for 72 h at 30 °C in 96-well plates, with biological quadruplicates. Cell growth was determined by measuring OD_600_, followed by staining with Nile blue sulfate (20 µL of a 2.2 g L⁻^1^ aqueous solution). Fluorescence was measured at excitation and emission wavelengths of 490 and 590 nm, respectively. Relative PHA production was calculated by normalizing the difference between fluorescence values measured (before and after staining) to OD_600_. PHA production was compared using one-way ANOVA followed by Tukey’s test, with a significance level of 0.05. *Escherichia coli* ATCC 25922 grown in glucose-supplemented mineral medium was used as a negative control. The isolate exhibiting the highest PHA production was selected for PHA characterization by gas chromatography–mass spectrometry (GC/MS).

For PHAs monomer characterization, polymer extraction and derivatization were carried out according to the method described by Oehmen et al. (2005) using biomass of isolate TA18 grown in MSM10 and sodium benzoate as an internal standard. Analysis of derivatized samples was performed using gas chromatography-mass spectrometry (GC/MS). Chromatographic peaks were assigned to the best-matching compound in the NIST14 database regardless of compound class, enabling identification of both PHA monomers and other lipid-related compounds present in the derivatized biomass (see SM4).

## Results and discussion

### Isolation and identification of TPA-utilizing bacteria

A total of 42 bacterial colonies were isolated from the enrichment cultures in MM5 incubated at 30 °C. No increase in turbidity was observed at the end of the enrichment performed at 50 °C. The absence of microbial growth at 50 °C indicates that TPA-degrading populations in the tested compost are predominantly mesophilic, which is consistent with the absence of reports of both thermotolerant and thermophilic strains in TPA upcycling studies (Somiza et al. 2026). The isolates obtained were sequentially designated as TA1 to TA42 (fully available in Table S2). Among them, seven isolates (TA18, TA21, TA27, TA33, TA34, TA38, and TA39) degraded more than 90% of TPA within 72 h; therefore, they were selected for subsequent experiments. The remaining isolates within the screening were not identified or further characterized.

**Fig. 1:**
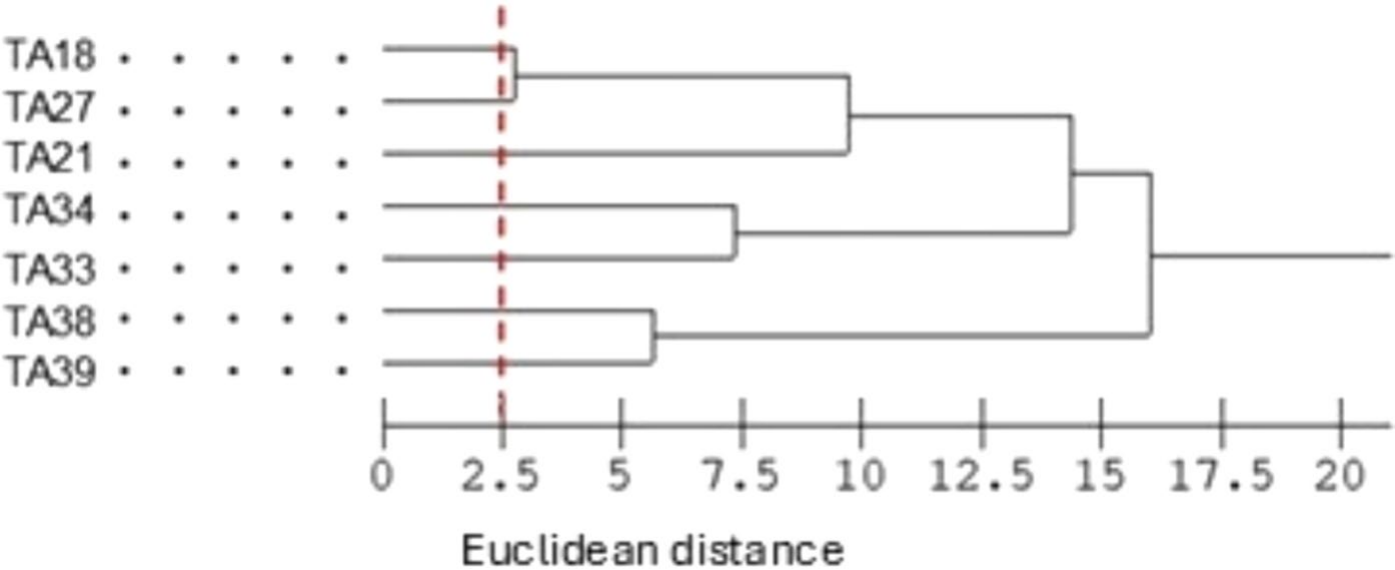
Euclidean distance dendrogram of fatty acid profiles from the isolates generated using the Microbial Identification System (MIDI)

**Fig. 2:**
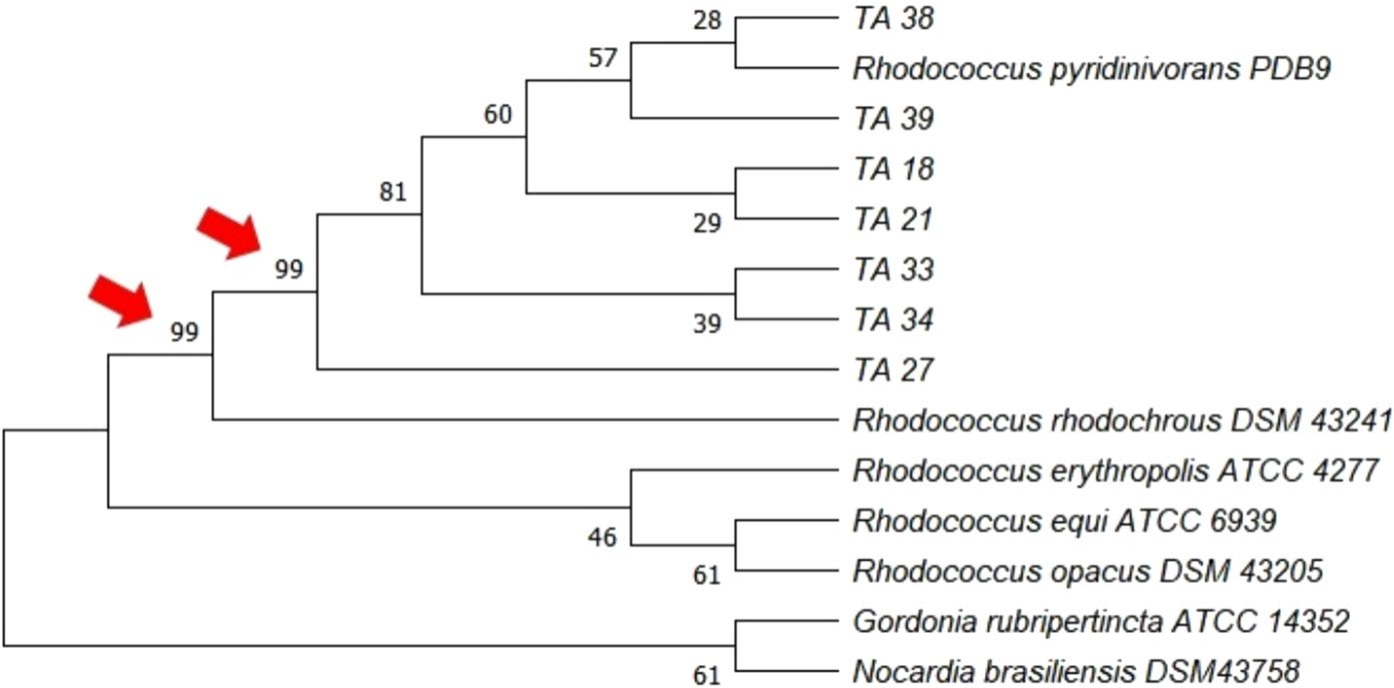
Phylogenetic tree based on 16S rRNA gene sequencing, comparing the isolates with other type strains from the respective genera identified by BLASTn. Node values indicate bootstrap support inferred using the maximum-likelihood method and the general time reversible model with 10,000 bootstraps. *Gordonia rubripertincta* and *Nocardia brasiliensis* were used as outgroup to root the tree

The seven isolates were considered distinct strains based on Euclidean distances among the fatty acid profiles generated by the MIDI system (Fig. 1). Furthermore, BLASTn analysis of the partial 16S rRNA gene sequences indicated *Rhodococcus pyridinivorans* as the closest match for all the selected isolates. Although 16S rRNA gene sequencing alone is insufficient for species-level resolution, high bootstrap values (Fig. 2, arrows) support placement of the strains within the genus *Rhodococcus*. Thus, the isolates were classified as *Rhodococcus*.

The classification of all seven strains within the genus *Rhodococcus* is consistent with the known capacity of this group to assimilate aromatic compounds and xenobiotics (Ivshina et al. 2022), and with the characterization of *Rhodococcus jostii* RHA1 as a model organism for TPA catabolism (Hara et al. 2007). Beyond degradative capacity, members of this genus are recognized producers of biotechnologically relevant compounds, including biosurfactants, pigments, siderophores, polyhydroxyalkanoates (PHAs), and triacylglycerols (TAGs) (Cappelletti et al. 2020), providing a rational basis for investigating their biosynthetic potential using TPA as the carbon source.

### Growth curves and TPA consumption by strains

**Fig. 3:**
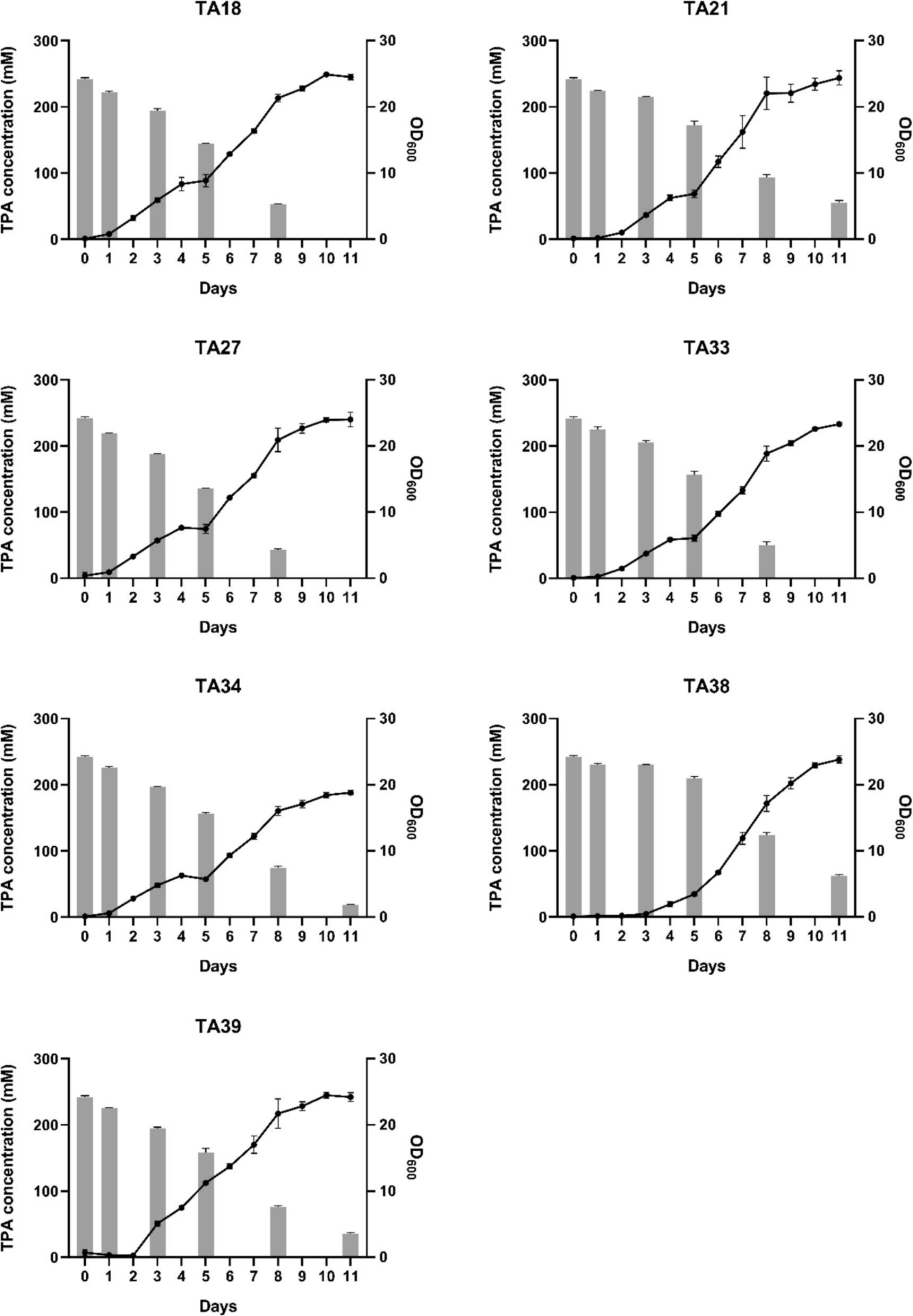
Growth of isolates in MM containing 240 mM TPA as the sole carbon source. Initial OD600 was set to 0.1 for all strains. Data points represent the mean OD_600_ values. Bars indicate TA concentration in the culture supernatant, quantified on days 1, 3, 5, 8, and 11. All analyses were performed in triplicate, and error bars indicate the standard deviation of the corresponding means

The growth performance on MSM40 highlights the stress tolerance and robustness often attributed to the *Rhodococcus* group. Based on medium composition, MSM40 is estimated to impose an osmolality of ~ 1.2 Osm, approximately three times the osmolality of Lysogeny Broth (~ 0.41 Osm). Although the high concentrations of NaOH and HEPES employed generated substantial osmotic and ionic stress, all strains grew on minimal medium and reached high cell densities. OD_600_ measurements indicated an apparent stabilization of growth after 11 d, reaching a final OD_600_ of approximately 24 for most isolates (Fig. 3).

This trait is particularly relevant for industrial applications, where harsh process conditions frequently restrict the use of promising microorganisms, and osmotic stress is a common constraint (Kuang et al. 2024). Even under these conditions, three isolates completely degraded 240 mM TPA within 11 d or less (TA18, TA27, and TA33). The complete consumption of 240 mM TPA by these isolates surpasses the highest concentration previously reported for wild-type strains, in which *Rhodococcus erythropolis* completely consumed 120 mM TPA within 84 h under optimized conditions supplemented with yeast extract (Maurya et al. 2024). The present results were obtained in strictly minimal medium under substantial osmotic stress, suggesting that the isolated strains display greater robustness under the osmotically and nutritionally challenging conditions employed here. For comparison, the highest TPA concentration employed in engineered strain studies was also 120 mM, used by Werner et al. (2021) with *Pseudomonas putida* KT2440 to produce β-ketoadipate, though complete TPA consumption was not achieved in that setup.

The NaOH-mediated solubilization of TPA employed in MSM40 generates Na₂TPA as the predominant dissolved species, which is also produced during alkaline PET hydrolysis (Barnard et al. 2021), thus providing a point of contact between the cultivation conditions used here and depolymerization-derived substrate streams. For reference, Anglou et al. (2024) maintained Na₂TPA below 476 mM (~ 10 wt%) to ensure complete solubilization of the hydrolysate, suggesting that only a two-fold dilution would be needed to use such streams directly as feedstock. This integration would also bypass some downstream steps required for TPA purification, such as acidification, precipitation, filtration, and neutralization, while enabling the biosynthesis of products with higher added value than recycled TPA, which could help reduce costs and improve overall process economics. Although such streams would also contain ethylene glycol and other co-products, the ability of these strains to grow and completely consume TPA under osmotically challenging conditions in minimal medium represents a relevant step toward the biological integration of PET hydrolysis and upcycling. Additionally, the high-density cultures achieved by the strains in these conditions support their physiological robustness and potential for future process development, such as process scale-up.

### Bioproducts assays

Under all tested conditions, no extracellular biosurfactants were detected in the cell-free supernatants. However, visible changes at the oil–water interface were observed in cultures grown on n-hexadecane and soybean oil, suggesting changes in interfacial tension. Based on this observation, we hypothesized that the biosurfactant was cell-associated rather than released into the medium. To test this, cell pellets from cultures grown on n-hexadecane and soybean oil were subjected to the oil-spreading assay, yielding positive results. This phenomenon has been reported for several *Rhodococcus* strains and may provide ecological advantages by increasing cell surface hydrophobicity, thereby facilitating adhesion to hydrophobic substrates (Sharma et al. 2021). In contrast, no biosurfactant activity was detected in cultures grown on TPA, and further characterization was therefore considered beyond the scope of this study.

**Fig. 4:**
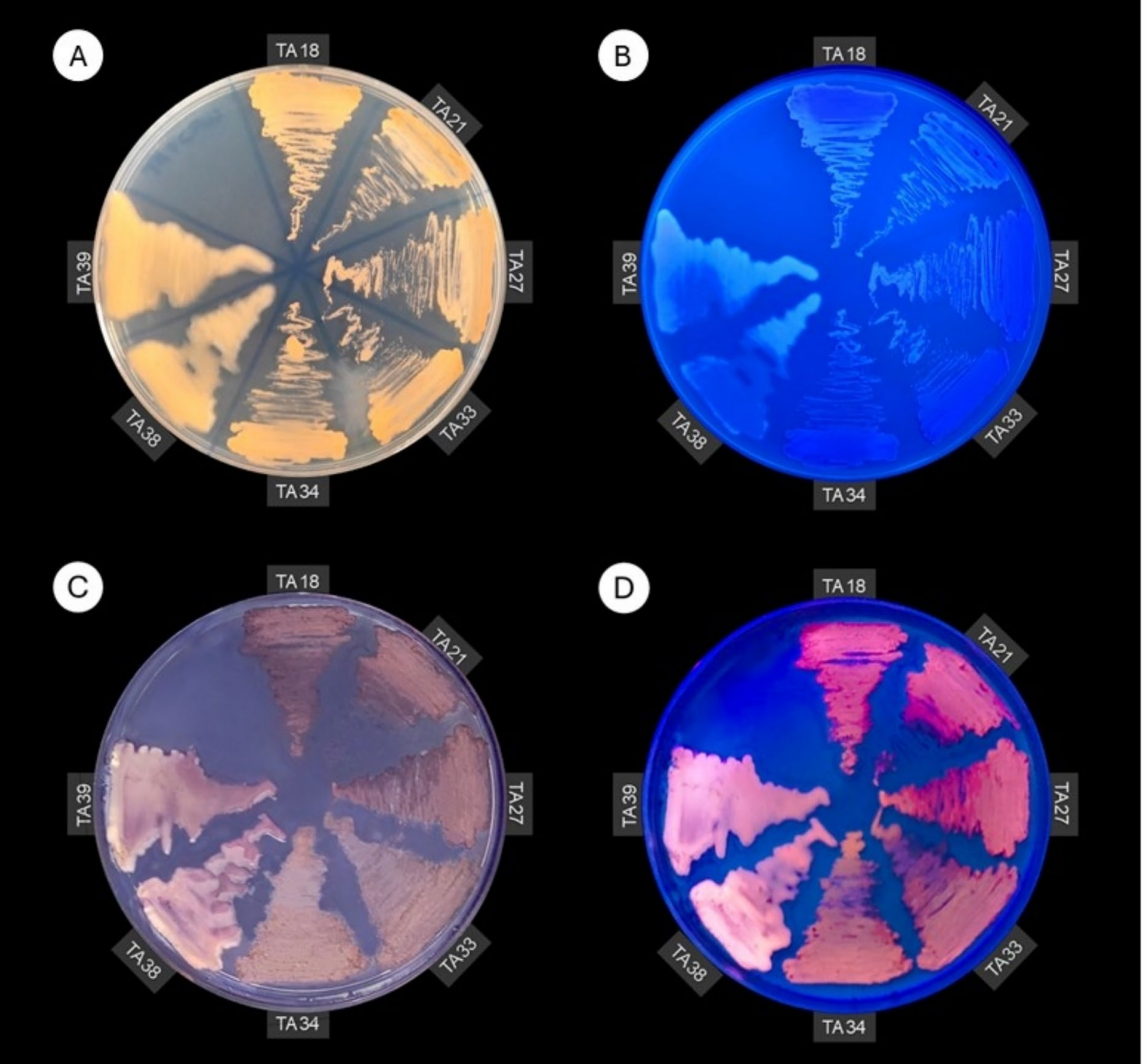
EPS and PHA production assays. (**A**, **B**) MSM10 supplemented with 0.2 g L^−1^ calcofluor. (**C**, **D**) MSM10 after addition of Nile Blue solution. (**A**, **C**) Plates under white light. (**B**, **D**) Plates under ultraviolet light (365 nm)

In addition, all selected isolates exhibited white-blue fluorescence under UV light when grown in the presence of calcofluor white, indicating the production of EPS containing β-linked polysaccharides when TPA was supplied as the sole carbon source (Fig. 4B). Although EPS formation has been reported in *Rhodococcus* under other cultivation conditions (Krivoruchko et al. 2026), our results show that this phenotype is also maintained during growth exclusively on TPA, indicating that carbon derived from TPA is directed not only to biomass formation but also to extracellular polysaccharide biosynthesis. This broadens the range of bioproducts associated with TPA assimilation in *Rhodococcus*. Further compositional analyses will be required to determine the structure of the polymers produced from TPA.

**Fig. 5:**
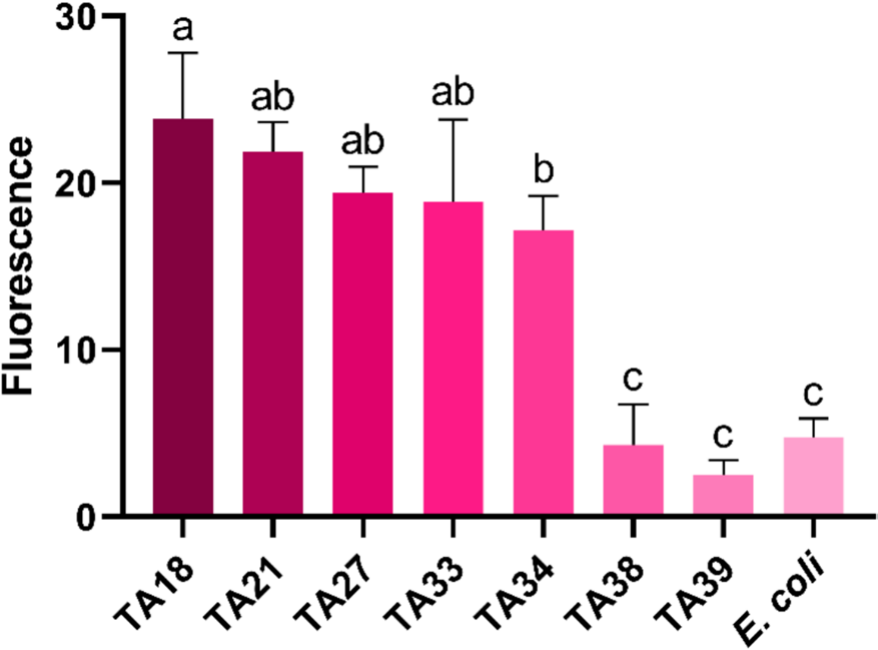
PHA production quantified by fluorescence, shown as the mean of four biological replicates with the corresponding standard deviations. *E. coli* ATCC 25922 grown in glucose was used as a negative control. Means followed by the same letter are not significantly different according to Tukey’s test at 5% probability

Lastly, the polyhydroxyalkanoate (PHA) accumulation plate assay indicated that most isolates exhibited strong fluorescence after staining, whereas isolates TA38 and TA39 showed low to no visible fluorescence (Fig. 4D). To compare PHA production among the isolates, the method proposed by Oshiki et al. (2011) was adapted to a 96-well plate format. Consistent with the plate-based assay, isolates TA38 and TA39 did not differ significantly from the negative control (Tukey’s test, p > 0.05) and were therefore excluded as PHA accumulators under the tested conditions (Fig. 5). Genomic analysis, such as whole-genome sequencing of the strains, would provide genetic context for the observed traits and may help explain the lack of PHA accumulation in TA38 and TA39. In contrast, isolate TA18 was the only strain assigned to the highest fluorescence group and differed significantly from isolate TA34, which showed the lowest mean fluorescence among PHA accumulators (Tukey’s test, p < 0.05); consequently, TA18 was selected for further characterization of lipid compounds by GC/MS.

### Characterization of lipid metabolites

**Fig. 6:**
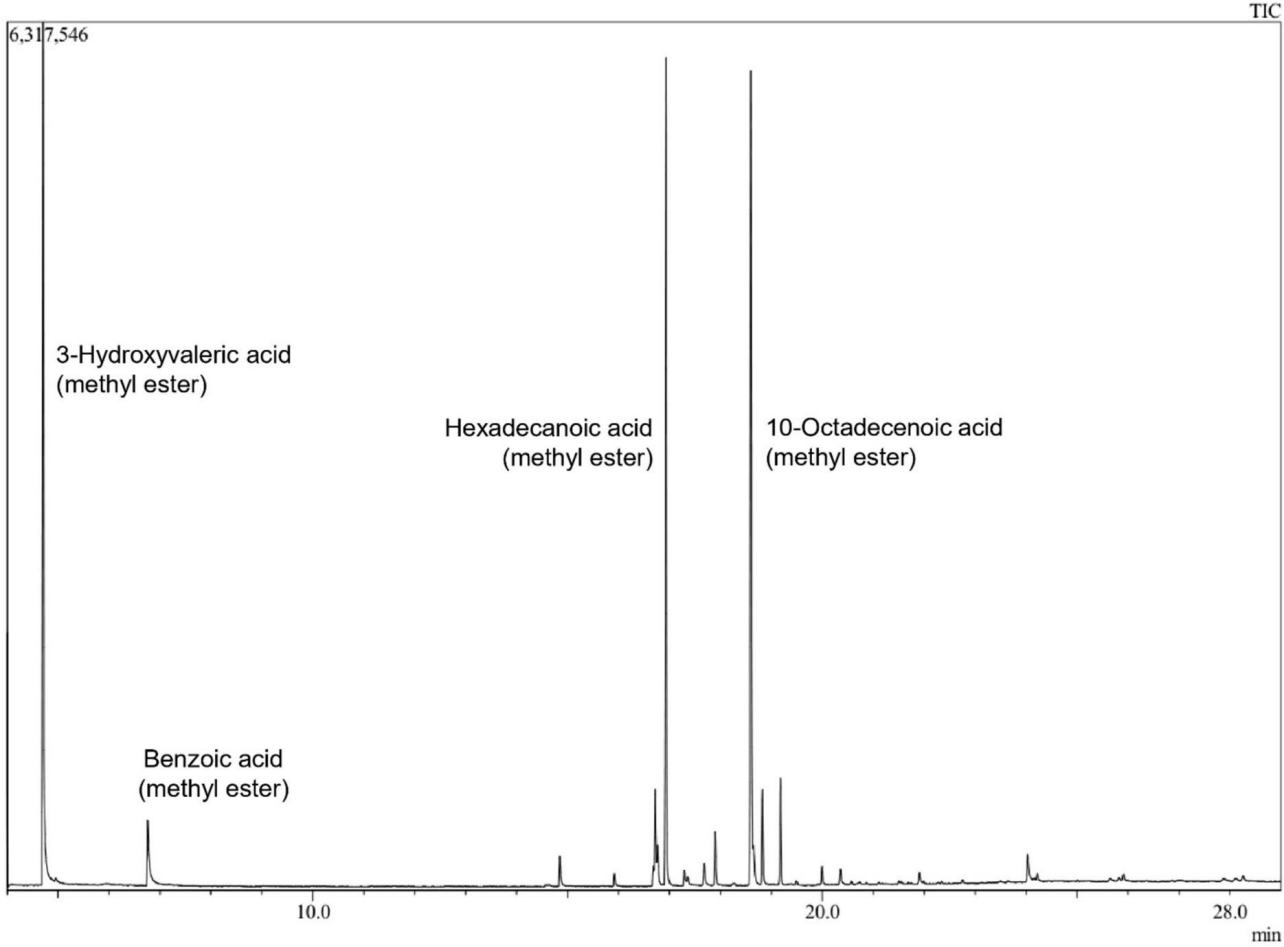
GC/MS chromatogram of methanolysis-derivatized TA18 biomass grown on MSM10, showing three mainpeaks identified by the highest similarity scores. Sodium benzoate was used as internal standard

GC/MS analysis of the derivatized biomass from isolate TA18 revealed three major peaks in the chromatographic profile (Fig. 6). These peaks were compared against the NIST14 mass spectral database and were identified as methyl esters of 3-hydroxyvalerate (3HV), hexadecanoate (palmitate), and an unsaturated C18:1 fatty acid, with similarity indices of 88%, 97%, and 96%, respectively. Although the similarity index for 3-hydroxypentanoate (88%) did not reach the recommended 90% threshold, the fragmentation pattern was consistent with a 3-hydroxy fatty acid methyl ester, and PHA accumulation was independently supported by Nile blue fluorescence assays. The C18:1 peak matched to 9-octadecenoic (oleic) and 10-octadecenoic (iso-oleic) acid with identical scores (96%); as GC/MS could not resolve the isomerism, we refer to it as a C18:1 fatty acid. Confirmation of monomer composition by FTIR or NMR represents a necessary next step for this finding, as pure poly(3-hydroxyvalerate) (PHV) production from TPA as the sole carbon source has not been previously described in *Rhodococcus*. The only prior report of pure PHV in this genus involved *Rhodococcus ruber* grown on five-carbon organic acids (Haywood et al. 1991).

Beyond 3HV, the chromatographic profile also revealed the accumulation of fatty acids in TA18, composed mainly of palmitic acid and a monounsaturated C18:1 fatty acid. Since both PHA monomer analysis and fatty acid identification rely on methanol transesterification, the same derivatized samples yielded evidence for both compound classes, supporting the validity of these identifications. In the chromatographic profile, the relative areas of the C16 and C18 peaks (23.5% and 27.6%, respectively) were comparable to that of the 3-HV peak (25.8%), collectively accounting for over 75% of the total signal. The precise nature of these lipids remains to be determined, as lipid fractionation prior to derivatization would be required to distinguish storage lipids from structural membrane components.

## Conclusion

The isolation of seven *Rhodococcus* strains from compost enrichment cultures, three of which completely consumed 240 mM TPA under osmotically challenging minimal medium conditions, demonstrates that environmental microbial diversity remains an underexplored source of robust TPA-degrading candidates. The biosynthetic profile of strain TA18 establishes that a single wild-type isolate can direct aromatic substrate carbon into multiple biotechnologically relevant compound classes, broadening the product scope of TPA bioconversion beyond what has been previously characterized. These strains provide a foundation for developing biological upcycling routes for PET waste, with performance on real depolymerization streams and metabolite quantification as the critical next steps.

## Supplementary Information

Below is the link to the electronic supplementary material.

Supplementary file1 (DOCX 27 KB)

## Data Availability

No datasets were generated or analysed during the current study.
