# Supplementary material for: High-load terephthalic acid degradation and diverse bioproduct formation by novel Rhodococcus strains
Source: Biotechnol Lett. 2026 May 13;48(3):68. doi: 10.1007/s10529-026-03739-z (PMC13171638; doi:10.1007/s10529-026-03739-z)
Supplement: Supplementary file 1 — Supplementary file1 (DOCX 27 KB) [file 10529_2026_3739_MOESM1_ESM.docx]

High-load terephthalic acid degradation and diverse bioproduct formation by novel *Rhodococcus* strains

Biotechnology Letters

Caio Issamu Somiza^1^*, Nívea Moreira Vieira^1^, Alex Gazolla de Castro^1^, Isabela Pereira da Silva Bento^2^, Kleryton Luiz Alves de Oliveira^3^, Lívia Moreira Couto^1^, Jomar de Lima Barros^1^, Camila de Souza Vieira^1^, Wendel Batista da Silveira^1^, Renata Pereira Lopes Moreira^3^, Marcos Rogério Tótola^1^

^1^Department of Microbiology, Federal University of Viçosa, Viçosa, Brazil

^2^Department of General Biology, Federal University of Viçosa, Viçosa, Brazil

^3^Department of Chemistry, Federal University of Viçosa, Viçosa, Brazil

*corresponding author

Email of corresponding author: caio.somiza@gmail.com

Supplementary Information

Table S1. Culture media employed in this study.

| **Medium** | **Composition (g L^-1^)** |
| --- | --- |
| Tryptic Soy Broth (TSB) | Casein peptone (17.0), soy peptone (3.0), glucose (2.5), NaCl (5.0) and K_2_HPO_4_ (2.5) |
| Tryptic Soy Agar (TSA) | TSB with the addition of agar (15.0) |
| Mineral Salts Medium (MSM) | K_2_HPO_4_ (2.27), KH_2_PO_4_ (0.95), NH_4_Cl (0.5) and 2 mL of metals solution (Na_2_EDTA.2H2O (6.37), ZnSO_4_.7H_2_O (1.0), CaCl_2_.2H_2_O (0.5), FeSO_4_.7H_2_O (2.5), NaMoO_4_.2H_2_O (0.1), CuSO_4_.5H_2_O (0.1), CoCl_2_.6H_2_O (0.2), MnSO_4_.H_2_O (0.52) and MgSO_4_.7H_2_O (60.0)) |
| MSM5 | MSM with 30 mM of terephthalic acid |
| MSM10 | MSM with 60 mM of terephthalic acid |
| MSM40 | MSM with 240 mM of terephthalic acid |

Table S2. Isolates from enrichment cultures labels.

| **Temperature (°C)** | **Isolate ID** | **Substrate degradation ability** |
| --- | --- | --- |
| 30 | TA1 | + |
|  | TA2 | + |
|  | TA3 | - |
|  | TA4 | - |
|  | TA5 | ++ |
|  | TA6 | ++ |
|  | TA7 | ++ |
|  | TA8 | + |
|  | TA9 | ++ |
|  | TA10 | + |
|  | TA11 | - |
|  | TA12 | - |
|  | TA13 | ++ |
|  | TA14 | - |
|  | TA15 | ++ |
|  | TA16 | ++ |
|  | TA17 | - |
|  | TA18 | +++ |
|  | TA19 | + |
|  | TA20 | ++ |
|  | TA21 | +++ |
|  | TA22 | - |
|  | TA23 | - |
|  | TA24 | - |
|  | TA25 | - |
|  | TA26 | - |
|  | TA27 | +++ |
|  | TA28 | + |
|  | TA29 | ++ |
|  | TA30 | ++ |
|  | TA31 | - |
|  | TA32 | ++ |
|  | TA33 | +++ |
| 30 | TA34 | +++ |
|  | TA35 | - |
|  | TA36 | ++ |
|  | TA37 | + |
|  | TA38 | +++ |
|  | TA39 | +++ |
|  | TA40 | - |
|  | TA41 | - |
|  | TA42 | - |
| 50 | - | - |

The isolates were classified according to relative substrate consumption after 72 h: (−) consumption below 10%; (+) consumption between 10 and 50%; (++) consumption between 50 and 90%; and (+++) consumption above 90%.

Table S3. Accession number for strains’ 16S rRNA sequences.

| **Strain** | **GenBank Accession Number** |
| --- | --- |
| *Rhodococcus* sp. LBBMA TA18 | PX886161 |
| *Rhodococcus* sp. LBBMA TA21 | PX886162 |
| *Rhodococcus* sp. LBBMA TA27 | PX886163 |
| *Rhodococcus* sp. LBBMA TA33 | PX886164 |
| *Rhodococcus* sp. LBBMA TA34 | PX886165 |
| *Rhodococcus* sp. LBBMA TA38 | PX886166 |
| *Rhodococcus* sp. LBBMA TA39 | PX886167 |

*Supplementary Methods 1 – Enrichment cultures and terephthalic acid quantification*

Enrichment cultures were prepared using mineral salts medium supplemented with 30 mM TA (MSM5). All media, whose composition is available at Table S1, were adjusted to an initial pH of 7.0. The inoculum was composed of 20 g of eucalyptus leaf and manure compost in 200 mL of saline solution, and each flask was added with 10% (v v^-1^) of this suspension. Cultures were incubated under orbital shaking at 200 rpm at 30 °C and 50 °C, to reflect the thermal variability of composting environments and to allow the enrichment of both mesophilic and thermotolerant microorganisms. Compost samples were collected in Viçosa, Minas Gerais, Brazil, in September 2023.

Cultures that grew were transferred to fresh medium using a 10% (v v^-1^) inoculum and this process was repeated three times. Following enrichment, cultures were serially diluted and plated onto MSM5 agar. Plates were incubated at the corresponding temperatures, and morphologically distinct colonies were isolated and purified on Tryptic Soy Agar (TSA). Pure isolates were subsequently cultivated in Tryptic Soy Broth (TSB) and stored at −80 °C in TSB supplemented with 25% (v v^-1^) glycerol at the Laboratório de Biotechnologia e Biodiversidade para o Meio Ambiente (LBBMA) culture collection, located at Federal University of Viçosa, Brazil.

*Supplementary Methods 2 – Quantification of terephthalic acid*

Residual TA quantification was quantified by high-performance liquid chromatography (HPLC) using a Phenomenex Kinetex C18 column (100 Å, 250 × 4.6 mm) coupled to a Shimadzu Prominence 20A liquid chromatograph equipped with a Shimadzu SPD-20A UV–Vis detector set at 250 nm. 1 µL of filtered culture supernatants were injected. The mobile phase consisted of 40% (v v^-1^) methanol in ultrapure water and was operated under isocratic conditions at a flow rate of 0.8 mL min⁻¹. The column temperature was maintained at 30 °C.

*Supplementary Methods 3 – Phylogenetic analysis*

Partial 16S rRNA gene sequencing were employed for bacterial identification. Genomic DNA was extracted using the GenElut Bacterial Genomic DNA Kit (Sigma-Aldrich, USA). Partial amplification of the 16S rRNA gene was performed by PCR using primers 27F (5’ AGAGTTTGATCCTGGCTCAG 3’) and 1492R (5’ GGTTACCTTGTTACGACTT 3’) under standard Taq polymerase condition. Sequences obtained were aligned using the MUSCLE algorithm, and phylogenetic inference was conducted using the Maximum Likelihood method and General Time Reversible model with 10,000 bootstraps. To compare the isolates with closely related species within the genus identified by BLASTn, 16S rRNA gene sequences from the type strains of *Rhodococcus pyridinivorans*, *R. rhodochrous*, *R. erythropolis*, *R. equi*, and *R. opacus* were included. Sequence from the type strain of *Gordonia rubripertincta* and *Nocardia brasiliensis* were used as outgroup to root the resulting tree as they belong to Nocardiaceae. A total of 14 sequences were included in the phylogenetic analysis between the isolated strains and reference sequences. All sequencing analyses were performed in MEGA X.

*Supplementary Methods 4 – Characterization of PHAs produced from TA*

PHAs were characterized by GC–MS, performed using a Shimadzu QP2010 gas chromatograph coupled to a 5975I mass selective detector and equipped with an HP-5MS capillary column (30 m × 0.25 mm × 0.25 µm). The injector, interface, quadrupole, and ion source temperatures were set to 250 °C, 280 °C, 120 °C, and 250 °C, respectively. The oven temperature program started at 40 °C and was increased at a rate of 10 °C min⁻¹ to 280 °C, which was held for 5 min. Helium was used as the carrier gas at a flow rate of 1.2 mL min⁻¹. A volume of 1 µL of the derivatized sample was injected, with a solvent delay of 4 min. Electron impact ionization at 70 eV was employed, and mass spectra were acquired over an m/z range of 60–600. Compound identification was performed by comparison with the NIST 14 mass spectral library, and assignments were based on the highest similarity index.
